# Electronic cigarette use and periodontal tissue damage: a scoping review and an evidence-informed conceptual framework

**DOI:** 10.3389/fdmed.2026.1956724

**Published:** 2026-09-21

**Authors:** K. Kruthika, M. U. Aishwarya, Krapa Vaishnavi, Krithi P. Shetty, J. Divyadharshini

**Affiliations:** Nitte (Deemed to be University), AB Shetty Memorial Institute of Dental Sciences (ABSMIDS), Department of Oral & Maxillofacial Surgery, Mangalore, India

**Keywords:** electronic cigarettes, evidence-informed conceptual framework, periodontal disease, periodontal health, scoping review, vaping

## Abstract

**Background:**

Electronic cigarette (e-cigarette) use has increased worldwide and is often promoted as a safer alternative to conventional smoking. However, its effects on periodontal health remain incompletely understood because of heterogeneous findings. This scoping review aimed to synthesize evidence comparing periodontal health among electronic cigarette users, conventional cigarette smokers, and non-smokers and to develop an evidence-informed conceptual framework illustrating potential biological and clinical pathways linking electronic cigarette use to periodontal tissue changes.

**Methods:**

A scoping review was conducted according to PRISMA-ScR. PubMed, Embase, Scopus, and Google Scholar were searched for studies published between January 2015 and March 2025. Eligible studies comparing periodontal clinical outcomes and inflammatory biomarkers among electronic cigarette users, conventional smokers, and/or non-smokers were narratively synthesized, and an evidence-informed conceptual framework was developed.

**Results:**

Of 379 records identified, 10 studies met the eligibility criteria. Conventional cigarette smokers generally demonstrated greater periodontal destruction than electronic cigarette users. Electronic cigarette users frequently demonstrated intermediate periodontal findings, including increased probing depth, clinical attachment loss, plaque accumulation, and alterations in bleeding on probing and inflammatory biomarkers; however, findings varied across studies. Potential pathways involving oxidative stress, immune dysregulation, vascular alterations, impaired wound healing, and oral microbiome alterations were identified.

**Conclusions:**

The available evidence suggests that electronic cigarette use may be associated with adverse periodontal changes compared with non-smoking, although findings are heterogeneous. The proposed evidence-informed conceptual framework integrates potential biological and clinical pathways and may guide future mechanistic, longitudinal, and multicenter research and periodontal risk assessment.

## 1 Introduction

Periodontal disease is a chronic inflammatory condition characterized by the progressive destruction of the gingiva, periodontal ligament, cementum, and alveolar bone, ultimately leading to tooth loss if left untreated. Although microbial biofilm initiates the disease, its progression is strongly influenced by host immune responses and modifiable environmental risk factors (1, 2). Conventional cigarette smoking is one of the most established modifiable risk factors for periodontal disease, promoting periodontal destruction through oxidative stress, immune dysregulation, vascular alterations, and impaired wound healing, and is associated with greater clinical attachment loss, deeper periodontal pockets, and poorer periodontal outcomes than those observed in non-smokers (1, 2).

Electronic cigarettes (e-cigarettes), also known as electronic nicotine delivery systems (ENDS), have emerged as widely used alternatives to conventional tobacco smoking (3). These devices generate an aerosol containing nicotine, propylene glycol, vegetable glycerin, flavoring agents, and other chemical constituents (3). Although e-cigarettes avoid many combustion-related toxicants generated by conventional cigarettes, experimental and clinical evidence suggests that their aerosols may induce oxidative stress, inflammatory responses, cellular dysfunction, and alterations in the oral microbiome, potentially affecting periodontal tissues (3, 4).

Several clinical studies have evaluated periodontal health among electronic cigarette users, conventional cigarette smokers, and non-smokers using parameters such as probing depth, clinical attachment loss, plaque index, gingival index, bleeding on probing, and inflammatory biomarkers (4–9). Across individual studies, electronic cigarette users frequently demonstrated periodontal findings that were intermediate between those observed in conventional cigarette smokers and non-smokers (4, 8–11). However, the magnitude and consistency of these associations varied according to study design, participant characteristics, vaping exposure, smoking history, and periodontal outcome measures (4, 11–13). Recent systematic reviews and meta-analyses have likewise reported heterogeneous findings, with conventional cigarette smokers generally showing poorer periodontal outcomes, while differences between electronic cigarette users and non-smokers have not been consistently demonstrated across all periodontal parameters (14–17).

Previous evidence syntheses have examined the periodontal effects of electronic cigarette use, and recent systematic reviews and meta-analyses have further expanded the evidence base while highlighting substantial heterogeneity and methodological limitations (14–17). However, the available literature remains heterogeneous across clinical outcomes, biological mechanisms, and study designs. A structured synthesis that integrates these domains and translates the reported findings into an evidence-informed conceptual framework may therefore provide additional value. Accordingly, the present review sought not only to map the available comparative periodontal evidence but also to integrate the reported biological mechanisms and potential pathways underlying the periodontal effects of electronic cigarette exposure.

This scoping review aimed to systematically map the available evidence comparing periodontal health among electronic cigarette users, conventional cigarette smokers, and non-smokers; synthesize the reported clinical and inflammatory findings; and integrate these findings into an evidence-informed conceptual framework illustrating potential biological and clinical pathways linking electronic cigarette use with periodontal tissue changes. Given the heterogeneity of the available evidence, the framework is intended as a hypothesis-generating model to organize current knowledge, support clinical risk assessment, and guide future mechanistic and longitudinal research rather than establish causal relationships.

## 2 Materials and methods

### 2.1 Study design

A scoping review was conducted to systematically map the available evidence regarding the effects of electronic cigarette (e-cigarette) use on periodontal health. The review was designed to address the following research question: “What is the impact of electronic cigarette use on periodontal health compared with conventional cigarette smokers and non-smokers?” The review was conducted in accordance with the Preferred Reporting Items for Systematic Reviews and Meta-Analyses Extension for Scoping Reviews (PRISMA-ScR) guidelines to ensure transparent and comprehensive reporting.

### 2.2 Search strategy

A comprehensive electronic literature search was conducted in PubMed, Embase, Scopus, and Google Scholar to identify relevant studies published between January 2015 and March 2025. Only English-language articles were considered.

The search strategy combined Medical Subject Headings (MeSH) and free-text keywords related to electronic cigarette use and periodontal health. The principal search terms included electronic cigarette, e-cigarette, vaping, electronic nicotine delivery systems (ENDS), periodontal disease, periodontitis, periodontal health, gingivitis, probing depth, and clinical attachment loss. These terms were combined using the Boolean operators AND and OR to maximize search sensitivity and identify all relevant studies. The reference lists of eligible articles were also manually screened to identify additional relevant publications.

### 2.3 Study selection

Two reviewers independently screened the retrieved records. Following removal of 29 duplicate records, 350 records were screened based on their titles and abstracts, of which 300 were excluded for reasons including irrelevant topics, non-clinical study design, review/editorial publication type, *in vitro* study design, or conference abstracts. The remaining 50 reports were sought for retrieval. Of these, 25 reports could not be retrieved because the full text was unavailable (*n* = 17) or could not be obtained despite retrieval attempts (*n* = 8). The remaining 25 full-text reports were assessed for eligibility, of which 15 were excluded because they did not meet the predefined eligibility criteria (*n* = 9) or contained insufficient data (*n* = 6). Ultimately, 10 studies were included in the review. Any disagreements between the two reviewers were resolved through discussion and consensus. No formal inter-reviewer agreement statistics were calculated. The study selection process is presented in the PRISMA-ScR flow diagram (Figure 1).

**Figure 1:**
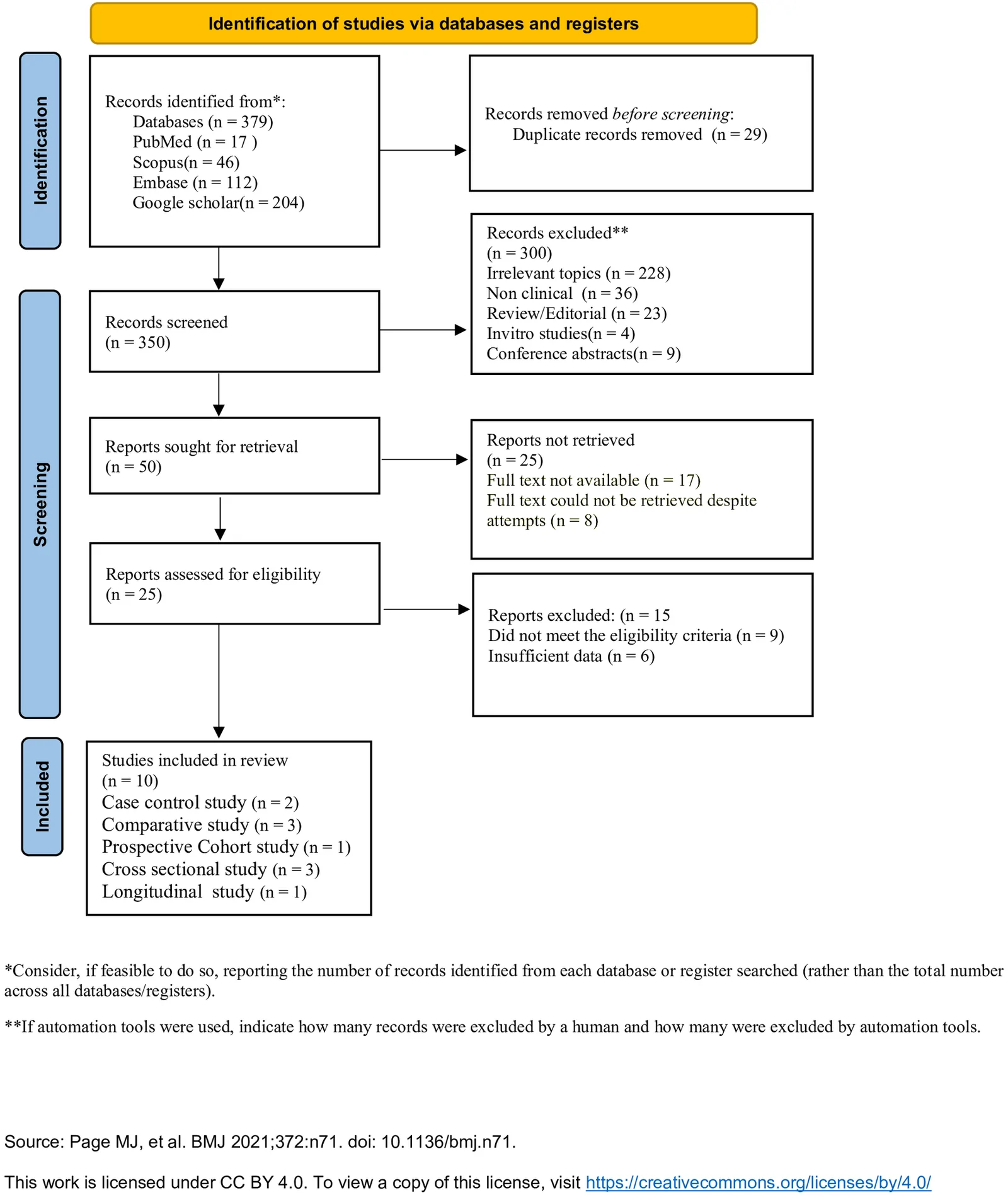
PRISMA-ScR flow diagram of the study selection process.

### 2.4 Eligibility criteria

#### 2.4.1 Inclusion criteria

Studies were included if they:

- Were original human clinical research;
- Employed observational study designs, including cross-sectional, comparative, case-control, cohort, prospective cohort, or longitudinal studies;
- Evaluated periodontal health among electronic cigarette users, conventional cigarette smokers, and/or non-smokers;
- Reported clinical periodontal parameters, inflammatory biomarkers, and/or microbiological outcomes; and
- Were published in English between January 2015 and March 2025.

#### 2.4.2 Exclusion criteria

Studies were excluded if they:

- Were systematic reviews, narrative reviews, editorials, letters, conference abstracts, or case reports;
- Were *in vitro* or animal studies;
- Did not evaluate periodontal outcomes associated with electronic cigarette use;
- Included participants with systemic conditions known to substantially influence periodontal health, such as uncontrolled diabetes mellitus, immunocompromised conditions, or pregnancy; or
- Were published in languages other than English.

### 2.5 Data extraction

Data were independently extracted by two reviewers using a standardized data-charting form. The extracted variables included the author, year of publication, country, study design, sample size, participant characteristics, smoking and vaping exposure, periodontal clinical parameters, inflammatory biomarkers, microbiological findings, and the key findings of each study.

### 2.6 Data synthesis

Owing to substantial heterogeneity in study design, participant characteristics, vaping exposure, outcome measures, and reported periodontal parameters, quantitative synthesis was not considered appropriate. Therefore, the findings were synthesized using a narrative thematic approach to identify recurring patterns in periodontal clinical outcomes, inflammatory biomarkers, and biological mechanisms across the included studies. Based on the narrative synthesis, an evidence-informed conceptual framework was developed to illustrate the potential biological pathways linking electronic cigarette use to periodontal tissue damage (Figure 2). The framework is intended as a hypothesis-generating conceptual model rather than a validated mechanistic pathway.

**Figure 2:**
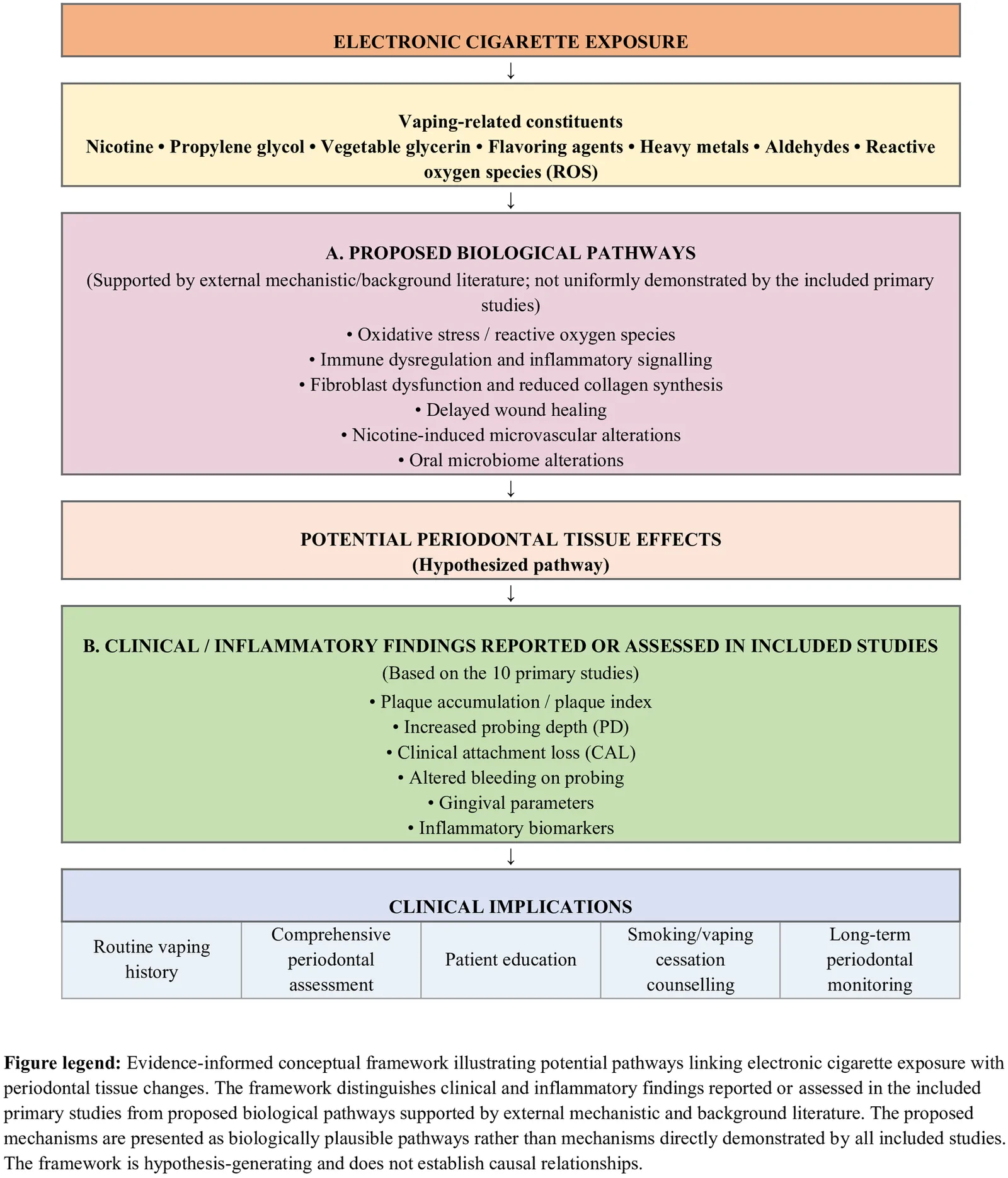
Evidence- informed conceptual framework illustrating the potential pathways linking electronic cigarette use to periodontal tissue damage.

### 2.7 Critical appraisal

In accordance with the objectives of a scoping review and the recommendations of the PRISMA-ScR guidelines, a formal methodological quality assessment or risk-of-bias evaluation of the included studies was not performed. The purpose of this review was to comprehensively map and synthesize the available evidence rather than determine the certainty of evidence, estimate pooled effect sizes, or evaluate intervention effectiveness.

## 3 Results

### 3.1 Study selection

The literature search identified 379 records from four electronic databases: PubMed (*n* = 17), Embase (*n* = 112), Scopus (*n* = 46), and Google Scholar (*n* = 204). After removal of 29 duplicate records, 350 unique articles remained for title and abstract screening. Of these, 300 records were excluded because they were unrelated to the review topic, were review articles, editorials, conference abstracts, *in vitro* or animal studies, or did not evaluate periodontal outcomes associated with electronic cigarette use. Fifty reports were sought for retrieval; however, 25 could not be retrieved, including 17 for which the full text was unavailable and 8 despite retrieval attempts. The remaining 25 reports were retrieved and assessed for eligibility. Following full-text assessment, 15 studies were excluded because they did not meet the eligibility criteria or lacked sufficient outcome data. Consequently, 10 studies were included in the final scoping review. The study selection process is illustrated in the PRISMA-ScR flow diagram (Figure 1).

### 3.2 Characteristics of the included studies

The characteristics of the included studies are summarized in Table 1. The studies were published between 2017 and 2025 and were conducted in Saudi Arabia (*n* = 4), Kuwait (*n* = 2), Egypt (*n* = 1), Malaysia (*n* = 1), Turkey (*n* = 1), and the United States (*n* = 1). The included study designs comprised three comparative studies, three cross-sectional studies, two case-control studies, one prospective cohort study, and one longitudinal study.

**Table 1:** Demographic data.

Study	Year	Country	Study design	Sample size	Gender		Mean age (year)	
					Male	Female		
Fawad Javed	2017	Saudi Arabia	Comparative study	94	94 T-CIG = 33 E-CIG = 31 NS = 30	0	T-CIG = 41.3 ± 2.8 E-CIG = 37.6 ± 2.1 NS = 40.7 ± 1.6	
N.M Kamal et al	2022	Egypt	Comparative study	150	86 T-CIG = 28 (56%) E-CIG = 32 (64%) NS = 26 (52%)	64 T-CIG = 22 (44%) E-CIG = 18 (36%) NS = 24 (48%)	T-CIG = 29.23 ± 5.83 E-CIG = 29.36 ± 8.01 NS = 28.33 ± 8.23	
Almazyad et al	2024	Saudi Arabia	Cross sectional	60	60(NR) T-CIG = 30 E-CIG = 30		>25 years; mean age −36.6 years	
Alnufaiy et al	2025	Saudi Arabia	Cross sectional study	53	53 E-CIG = 25 NS-28	0	E-CIG = 24.52 ± 2.29 NS = 37 ± 1.4	
Hasan et al	2024	Malaysia	Cross sectional study	144	144 T-CIG = 48 E-CIG = 48 N.S = 48	0	T-CIG = 34.83 ± 6.33 E-CIG = 31.77 ± 6.92 N.S = 34.48 ± 7.18	
Alkhalifah A.B. et al	2024	Kuwait	Prospective Cohort study	38	38 E-CIG = 20 NS = 18	0	18-25yrs ENDS users = 22.4 NS = 22.6	
Xu et.al	2021	United States	Longitudinal study	101	70%	30%	Males- T-CIG = 46.9 ± 10.1 E-CIG = 36.0 ± 9.8 NS = 28.8 ± 6.1	Females T-CIG = 46.6 ± 11.5 E-CIG = 38.7 ± 10.6 NS = 39.0 ± 14.1
					T-CIG = 31 E-CIG = 32 NS = 38			
F. Karaaslan et al	2020	Turkey	Comparative study	57	39	18	35.19 ± 2.23	
					T-CIG = 19 E-CIG = 19 Former Smokers = 19			
Ali et al	2022	Kuwait	Case control study	75	54	21	T-CIG = 52.6 ± 6.1 years E-CIG = 49.5 ± 2.3 years NS(with periodontitis) = 50.7 ± 2.2 years NS(without periodontits) = 48.1 ± 1.3 years	
					T-CIG = 15: 4 E-CIG = 12: 6 NS(with periodontitis) = 13: 6 NS(without periodontits) = 14: 5			
W L Ibraheem et al	2020	Saudi Arabia	Case control study	120	120 T-CIG = 30 E-CIG = 30 WATER-PIPE CIG = 30 NS = 30	0	T-CIG = 46.5 ± 5.3 E-CIG = 45.6 ± 3.6 WATER-PIPE CIG = 45.5 ± 4.4 NS = 43.8 ± 1.7	

T-CIG, traditional cigarette smokers; E-CIG, electronic cigarette users; NS, non-smokers; ENDS, electronic nicotine delivery systems; NR: not reported. Data are presented as reported in the original studies.

Sample sizes ranged from 38 to 150 participants. Most studies predominantly included male participants, whereas three studies recruited both male and female participants. The mean age of participants ranged from approximately 22 to 53 years. Most studies compared traditional cigarette smokers (T-CIG), electronic cigarette users (E-CIG), and non-smokers (NS), while one study additionally included waterpipe smokers. Demographic characteristics and study designs are summarized in Table 1.

### 3.3 Smoking and vaping characteristics

Smoking and vaping characteristics are summarized in Table 2. Conventional cigarette smoking was generally reported as the number of cigarettes smoked per day, whereas electronic cigarette exposure was described using diverse measures, including vaping sessions per day, puffs per session, tanks per day, or frequency of use.

**Table 2:** Comparison of frequency and duration of smoking between traditional cigarette smokers (T-CIG), e-cigarette smokers (E-CIG) and non-smokers).

Study	Sample size	T-cig		E-cig		Ns
		Frequency	Duration	Frequency	Duration	_
Favad Javed	94	1.33 ± 2.6 Times/day	5.4 ± 1.6 years	6.8 ± 0.8 times/day	2.2 ± 0.2 years	_
N.M Kamal et al	150	14.7 ± 2.5 times/day	5.2 ± 0.8 min/session	10.1 ± 1.4 times/day	7.8 ± 0.4 min/session	_
Almazya d et al	60	4-7 cigs per day	5-7 years	1-3 tanks/day	3-5 years	_
Alnufaiy et al	53	Not specified	Not specified	Not specified	432.6 ± 425.22	_
Hasan et al	144	13.10 ± 7.49 times/day	11.9 ± 6.35 years	Not specified	4.15 ± 2.76 years	_
Alkhalifah A B et al	38	Not specified	Not specified	6 puffs/session	19 years	_
Xu et al	101	13.5/day	Not specified	0.78/day	51.3 puffs/use	_
F Karaaslan et al	57	13.68 ± 3.67/day	13.95 ± 3.01 years	12.89 ± 2.54/day	12.11 ± 1.52 years	_
Ali et al.	75	Not specified	24.3 ± 0.7 years	25.1 ± 3.5 times/day	12.5 ± 0.8 years	_
W.I. Ibraheem et al	120	12.6 ± 1.5 times/day	18.3 ± 2.8 years	15.4 ± 0.3 times/day	6.4 ± 0.2 years	_

T-CIG, traditional cigarette smokers; E-CIG, electronic cigarette users; NS, non-smokers.

Among conventional cigarette smokers, reported smoking frequency ranged from 4 to 7 cigarettes per day to approximately 15 cigarettes per day, with smoking duration ranging from approximately 5 to 24 years. In contrast, electronic cigarette use demonstrated substantial variability in both frequency and duration. Reported vaping exposure ranged from 0.78 sessions per day to 25.1 sessions per day, whereas other studies described consumption as 1–3 tanks per day or 6 puffs per session. The reported duration of electronic cigarette use ranged from approximately 2 to 19 years, although several studies did not report exposure duration. Overall, considerable heterogeneity was observed in the measurement and reporting of vaping exposure.

### 3.4 Clinical periodontal findings

The clinical periodontal findings are presented in Table 3. Across the included studies, conventional cigarette smokers generally exhibited poorer periodontal clinical parameters than both electronic cigarette users and non-smokers.

**Table 3:** Comparison of clinical periodontal parameters between vaping/E-cigarette smokers, traditional cigarette smokers and Non-smokers.

Study	Periodontal parameters					
	PD (mm)	PI (mm)	CAL (mm)	GI (mm)	BOP	IL-1 *β*
Favad Javed	T-CIG = 29.3+-1.7 E-CIG = 5.1+-1.2 NS = 5.6+-0.8	T-CIG = 52.1+-6.6 E-CIG = 23.3+-3.4 NS = 21.4+-2.8	T-CIG = 2.1+-0.2 E-CIG = 1.1+-0.2 NS = 0.8+-0.1	-	T-Cig = 5.8+-0.8 E-Cig = 4.6+-2.9 NS = 27.5+-3.2	-
N M Kamal et al	-	-	-	-	-	Higher levels of IL-1 β in T-Cig than E-Cig
Almazyad et al	-	T-CIG = 5.92 E-CIG = 4.08	T-CIG = 3.44 E-CIG = 2.98	T-CIG = 1.79 E-CIG = 1.21	-	-
Alnufaiy et al	NS = 2.72 E-CIG = 4.10	NS = 41.00 E-CIG = 48.16	NS = 1.64 E-CIG = 2.72	-	NS = 34.03 E-CIG = 28.6	-
Hasan et al	T-CIG = 47.63% E-CIG = 36.79+-31.42 NS = 36.40+-24.85	-	T-CIG = 2.77+-0.64 E-CIG = 2.68+-0.66 NS = 2.42+-0.66	T-CIG = 11.79+-5.96 E-CIG = 16.15+-8.94 NS = 17.42+-17.2	-	-
Alkhalifah A B et al	E-CIG = 1.91+-0.3mm NS = 1.75+-0.2	E-CIG = 0.54+-0.2 NS = 0.43+-0.3	-	-	E-Cig = 20.2+-15.7 NS = 15.1+-11.8	-
Xu et al	T-CIG = 3.1 E-CIG = 3.0 NS = 2.7	-	T-CIG = 3.5 E-CIG = 2.8 NS = 2.2F	-	T-CIG = 61.5 E-CIG = 53.0 NS = 52.6	-
F. Karaaslan	T-CIG = 4.05+-0.64 E-CIG = 4.03+-0.66	T-Cig = 2.14+-0.41 E-Cig = 2.11+-0.37	T-CIG = 3.45+-0.71 E-CIG = 3.42+-0.67	T-CIG = 1.53+-0.29 E-CIG = 1.81+-0.30	-	-
Ali et al	T-CIG = 6.5+-0.2 E-CIG = 5.7+-0.2 3 = 6.1 4 = 1.2	T-CG = 3.1+-0.2 E-CIG = 2.5+-0.2 3-2.3 4-0.3	T-CIG = 8.4+-0.5 E-CIG = 7.1+-0.4 3 = 7.8 4 = 0.2	T-CIG = 0.9+-0.4 E-CIG = 1.05 + -0.03 3 = 3.3 4 = 0.5	-	-
W L Ibraheem et al	T-CIG = 4.6+-0.4 mm E-CIG = 4.2+-0.2 mm NS = 1.8+-0.08 mm WP CIG = 4.8+-0.6 mm	T-CIG = 43.5+-5.6% E-CIG = 40.1+-2.2% NS = 15.2+-2.1% WP-CIG = 46.7+-4.1%	T-CIG = 2.8+-0.3 mm E-CIG = 2.5+-0.2 mm NS = 0.4+-0.05 mm WP-CIG = 2.7+-0.4 mm	-	-	-

T-Cig, Traditional cigarette; E-Cig, Electronic cigarette; NS, Non-smokers; PD, Probing depth; PI, Plaque index; CAL, Clinical attachment loss; GI, Gingival index; BOP, Bleeding on probing; IL-1β, Interleukin-1 beta.

Greater probing depth, clinical attachment loss, and plaque accumulation among conventional cigarette smokers were reported by Javed et al., Almazyad et al., Hasan et al., Ali et al., and Ibraheem et al. Electronic cigarette users generally exhibited intermediate periodontal measurements, with values greater than those observed among non-smokers but lower than those reported for conventional cigarette smokers. Kamal et al. also reported higher salivary interleukin-1β levels among conventional cigarette smokers than electronic cigarette users, indicating a greater inflammatory response.

Some studies, however, reported different findings. Alnufaiy et al. observed greater probing depth, plaque index, and clinical attachment loss among electronic cigarette users than non-smokers. Similarly, Alkhalifah et al. reported higher probing depth, plaque index, and bleeding on probing among electronic cigarette users compared with non-smokers. The longitudinal study by Xu et al. reported greater clinical attachment loss among electronic cigarette users than non-smokers, with periodontal parameters increasing over time across all exposure groups. In contrast, Karaaslan et al. reported comparable probing depth, plaque index, and clinical attachment loss between conventional cigarette smokers and electronic cigarette users.

Findings related to gingival index and bleeding on probing were less consistent. Several studies reported lower bleeding on probing among conventional cigarette smokers despite greater periodontal destruction, whereas higher bleeding scores were frequently observed among non-smokers. Gingival index values also varied across studies, reflecting differences in inflammatory presentation among the study populations.

### 3.5 Overall evidence synthesis

Collectively, the included studies suggest that both conventional cigarette smoking and electronic cigarette use may be associated with adverse periodontal outcomes compared with non-smokers. Conventional cigarette smokers generally exhibited the greatest periodontal destruction across the reported clinical parameters, whereas electronic cigarette users frequently demonstrated intermediate periodontal findings. However, the direction and magnitude of periodontal differences between electronic cigarette users and non-smokers varied across studies, with some studies reporting poorer periodontal parameters among electronic cigarette users and others demonstrating more comparable findings.

Considerable heterogeneity was observed in study design, participant characteristics, exposure assessment, and reported periodontal outcomes, precluding quantitative synthesis. The integrated findings of the included studies were subsequently synthesized to develop an evidence-informed conceptual framework illustrating potential biological and clinical pathways through which electronic cigarette use may influence periodontal tissue health (Figure 2).

## 4 Discussion

### 4.1 Principal findings

This scoping review suggests that both conventional cigarette smoking and electronic cigarette use may be associated with adverse periodontal changes compared with non-smoking, although the magnitude and consistency of these associations vary across studies (4–13).

Conventional cigarette smokers generally demonstrated greater periodontal destruction than electronic cigarette users in several comparative studies (4, 7–11). Electronic cigarette users frequently exhibited intermediate periodontal findings relative to conventional cigarette smokers and non-smokers (4, 8–11). However, some studies reported greater periodontal alterations among electronic cigarette users compared with non-smokers, while others found more comparable periodontal parameters between exposure groups (5, 6, 12, 13).

The included primary studies reported differences in probing depth, clinical attachment loss, plaque accumulation, bleeding on probing, and inflammatory biomarkers among electronic cigarette users compared with non-smokers (4, 5, 8–13). However, the direction and magnitude of these findings varied across studies (5, 6, 12, 13). Recent systematic reviews and meta-analyses have similarly emphasized heterogeneity in periodontal outcomes and methodological limitations within the available evidence (14–17).

### 4.2 Biological mechanisms underlying vaping-associated periodontal damage

The adverse periodontal effects associated with electronic cigarette use are likely mediated through multiple interacting biological mechanisms. Although electronic cigarettes eliminate combustion-related toxicants found in conventional cigarettes, the generated aerosol contains nicotine, aldehydes, flavoring compounds, heavy metals, and reactive oxygen species, which may affect oral and periodontal tissues (3).

Experimental and clinical evidence suggests that these constituents may promote oxidative stress, impair fibroblast proliferation and collagen synthesis, dysregulate host immune responses, delay wound healing, and alter the oral microbiome (3, 5). Nicotine-induced vasoconstriction may further modify gingival blood flow and influence the clinical expression of gingival inflammation (3–5). These mechanisms provide biological plausibility for the periodontal alterations observed among electronic cigarette users, although a direct causal pathway has not been established (3, 5, 6).

Several included studies reported alterations in inflammatory biomarkers among electronic cigarette users, including interleukin-1β and other cytokines (8, 9, 12, 13). Although inflammatory activity appeared less pronounced than that associated with conventional cigarette smoking in several studies, findings varied across populations and outcome measures (8, 9, 13). Recent systematic reviews have similarly emphasized substantial heterogeneity and methodological limitations, making it premature to establish a definitive causal relationship between electronic cigarette exposure and periodontal tissue destruction (14–17).

### 4.3 Evidence-informed conceptual framework

Based on the synthesis of the included studies, an evidence-informed conceptual framework was developed (Figure 2) to illustrate potential biological and clinical pathways linking electronic cigarette use with periodontal tissue changes. The framework distinguishes between clinical and inflammatory findings directly reported or assessed in the ten included primary studies and biologically plausible mechanisms supported by external mechanistic and background literature.

The clinical and inflammatory findings represented in the included studies included changes in probing depth, clinical attachment loss, plaque accumulation, bleeding on probing, gingival parameters, and inflammatory biomarkers (4–13). In contrast, oxidative stress, immune dysregulation, inflammatory signalling, vascular alterations, fibroblast dysfunction, impaired collagen synthesis, delayed wound healing, and oral microbiome alterations are presented as proposed biological pathways that may contribute to periodontal tissue changes following electronic cigarette exposure (3). These mechanisms were not uniformly or directly evaluated in the ten included primary studies and therefore should not be interpreted as findings demonstrated by the present scoping review. The framework integrates these proposed biological pathways with the clinical and inflammatory findings identified in the included studies. As an evidence-informed synthesis of heterogeneous literature, the framework should be regarded as a hypothesis-generating conceptual model rather than a validated mechanistic or causal pathway. It is intended to organize current knowledge and guide future mechanistic and longitudinal research rather than establish causal relationships.

### 4.4 Clinical implications

The findings of this review have important implications for clinical practice. Oral healthcare professionals should routinely assess vaping history during periodontal examinations and consider electronic cigarette exposure as a potential periodontal risk indicator (4, 5, 9–17). The reduced bleeding on probing reported in some studies of nicotine users should be interpreted cautiously, as nicotine-related vascular effects may influence the clinical expression of gingival inflammation (3–5). Comprehensive periodontal assessment should therefore include multiple clinical parameters rather than relying solely on gingival bleeding. Patient education regarding the potential oral health effects of electronic cigarette use and appropriate smoking or vaping cessation counselling may also be incorporated into comprehensive periodontal care (18, 19).

### 4.5 Strengths and limitations

This review provides a comprehensive synthesis of current evidence comparing periodontal health among electronic cigarette users, conventional cigarette smokers, and non-smokers. The inclusion of studies from multiple countries and diverse observational study designs offers a broad overview of the available literature. Furthermore, integrating clinical periodontal findings with inflammatory biomarkers enabled the development of an evidence-informed conceptual framework summarizing potential biological pathways linking vaping with periodontal tissue changes (3–13).

Nevertheless, several limitations should be acknowledged. Most included studies were observational, limiting causal inference (4–13). Considerable heterogeneity existed regarding participant characteristics, electronic cigarette devices, nicotine concentrations, exposure duration, and periodontal outcome measures, precluding quantitative synthesis (4–6, 8–13). Smoking and vaping histories were largely self-reported in several studies, while potential confounding variables such as oral hygiene practices, systemic health conditions, socioeconomic status, and alcohol consumption were inconsistently controlled (4–6, 8–13). Evidence regarding microbiological changes and long-term periodontal treatment outcomes also remains limited (5–7, 14–17).

An additional limitation is the marked geographic concentration of the available evidence, with approximately 80% of the included studies conducted in the Middle East (4, 6–9, 11–13). This clustering may limit the generalizability of the findings, as patterns of electronic cigarette use, device characteristics, nicotine exposure, tobacco-use history, oral hygiene practices, and sociodemographic factors may vary across populations and healthcare settings. Consequently, the periodontal effects observed in the included studies may not be fully representative of populations from other geographic and cultural contexts. Future research should therefore prioritize multicenter, longitudinal studies involving geographically and demographically diverse populations to determine whether the observed periodontal effects are consistent across different demographic groups and to strengthen the external validity of the evidence.

## 5 Conclusion

This scoping review suggests that both conventional cigarette smoking and electronic cigarette use are associated with adverse periodontal changes compared with non-smokers. Although periodontal destruction among electronic cigarette users was generally less severe than that observed in conventional cigarette smokers, the available evidence suggests poorer periodontal health among electronic cigarette users than non-smokers, although findings were not consistent across all periodontal parameters. The included studies suggest that vaping may contribute to increased probing depth, clinical attachment loss, plaque accumulation, and altered inflammatory responses, although the magnitude of these effects varies across studies.

The evidence-informed conceptual framework developed from this review integrates the current understanding of the potential biological and clinical pathways linking electronic cigarette use to periodontal tissue damage. As an evidence-informed synthesis of the available literature, the framework should be regarded as a hypothesis-generating conceptual model rather than a validated mechanistic pathway. It may guide future mechanistic research, longitudinal investigations, and clinical risk assessment. Further high-quality prospective, multicenter studies using standardized exposure assessment are needed to clarify the long-term periodontal consequences of electronic cigarette use and strengthen future clinical recommendations for periodontal risk assessment and prevention.

## 6 Clinical significance

The findings of this scoping review suggest that electronic cigarette use may be associated with adverse periodontal changes compared with non-smokers, although the magnitude and consistency of these effects vary across studies. The proposed evidence-informed conceptual framework summarizes the potential biological and clinical pathways through which vaping may contribute to periodontal tissue changes and provides a basis for further investigation. Dental professionals should routinely assess vaping history, perform comprehensive periodontal examinations, provide appropriate smoking and vaping cessation counselling, and educate patients about the potential periodontal risks associated with electronic cigarette use.
